# The College of Nursing (Limited by Guarantee)

**Published:** 1921-01-15

**Authors:** 


### THE COLLEGE OF NURSING (Limited by Guarantee).

REPLIES TO THE REFERENDUM.

MEMBERS are asked to read what appears on this subject on page 368.

LONDON CENTRE.

(Secretary, Miss M. A. BOMPAS, 7 Henrietta Street, Cavendish Square, W. 1.)

THE COMING ELECTIONS.

TUESDAY, JANUARY 25, 8 P.M.—An important General Meeting of members of the London Centre will be held at Guy's Hospital to discuss the policy of the Centre with regard to the forthcoming election of Council members. Every member is asked to make arrangements at once to attend this meeting, and to be prepared to nominate members, having previously obtained their consent to stand, for election.

SESSIONAL LECTURES.

MONDAY, FEBRUARY 7, 8 P.M.—Lecture by Sir Robert Armstrong Jones on "The Relation between Mental Nursing and Hospital Nursing" at 7 Henrietta Street, W. 1.

FRENCH CONVERSATION AND DEBATING CLASSES.

A French Conversation Class is held every Tuesday afternoon, at 3 P.M., at the Club Room, and a French Class for elementary students every Friday, at 8 P.M. Members of the London Centre wishing to join either should communicate with Miss Bompas.

The Voice Training and Debating Class will begin on Wednesday, January 19, at 8 P.M., at the Club. Members of the London Centre wishing to join should communicate with Miss Bompas.

AN IMPORTANT CENTRE.

An interesting lecture on "Psychology in the Treatment of Shell-Shock" was given on Thursday, January 6, to the members of the London Centre by Professor Brown, of King's College. All College members resident in London are invited to join the London Centre, and thus put themselves in a position to attend the lectures and classes, which are much appreciated. The Centre now numbers 840 members.

EDINBURGH CENTRE.

WEDNESDAY, JANUARY 19, 3.30 P.M.—In the Nurses' Club, 8 Drumsheugh Gardens, a lecture by G. D. Mathewson, Esq., M.D., F.R.C.P.E., on "Dietetics in Disease." The lecture is open to members of the Club as well as to Centre members.

